# Ablation of Coactivator Med1 Switches the Cell Fate of Dental Epithelia to That Generating Hair

**DOI:** 10.1371/journal.pone.0099991

**Published:** 2014-06-20

**Authors:** Keigo Yoshizaki, Lizhi Hu, Thai Nguyen, Kiyoshi Sakai, Bing He, Chak Fong, Yoshihiko Yamada, Daniel D. Bikle, Yuko Oda

**Affiliations:** 1 Departments of Medicine and Endocrinology, University of California San Francisco and Veterans Affairs Medical Center San Francisco, San Francisco, California, United States of America; 2 Laboratory of Cell and Developmental Biology, National Institute of Dental and Craniofacial Research, National Institutes of Health, Bethesda, Maryland, United States of America; University of Tennessee, United States of America

## Abstract

Cell fates are determined by specific transcriptional programs. Here we provide evidence that the transcriptional coactivator, Mediator 1 (Med1), is essential for the cell fate determination of ectodermal epithelia. Conditional deletion of Med1 *in vivo* converted dental epithelia into epidermal epithelia, causing defects in enamel organ development while promoting hair formation in the incisors. We identified multiple processes by which hairs are generated in Med1 deficient incisors: 1) dental epithelial stem cells lacking Med 1 fail to commit to the dental lineage, 2) Sox2-expressing stem cells extend into the differentiation zone and remain multi-potent due to reduced Notch1 signaling, and 3) epidermal fate is induced by calcium as demonstrated in dental epithelial cell cultures. These results demonstrate that Med1 is a master regulator in adult stem cells to govern epithelial cell fate.

## Introduction

Postnatal cell fates are determined by adult stem cells residing in regenerative tissues. Understanding the mechanisms controlling cell fate is one of the fundamental goals in the field of cell biology. Dental epithelial stem cells (DE-SC) residing in the labial cervical loop (CL) continuously regenerate dental epithelia in the incisor throughout the life of the mouse. In contrast, dental epithelia in molars are not regenerated once molars are developed. DE-SCs share several characteristics with other adult stem cells in regenerative tissues such as slow division, discrete niche, and the ability to differentiate [1], [2]. DE-SCs are supported by a microenvironment within the CL, called the stem cell niche, that plays an important role in maintenance, proliferation, differentiation, and cell fate decisions during dental development [3] as observed in other self-renewing tissues [4]. DE-SCs are characterized by their signature molecules. Sox2 has been identified as a stem cell marker to maintain their lineages [5], [6]. DE-SCs give rise to all the dental epithelia including the inner and outer enamel epithelia (IEE, also called the inner dental epithelium [IDE], and OEE, respectively), the stellate reticulum (SR), the stratum intermedium (SI), and ameloblasts. Enamel matrix proteins are produced by ameloblasts at the secretory stage (Sec) and mineralized at the maturation (Mat) stage to form enamel. At the Mat stage, the dental papillary layer is invaded by the vasculature, which provides calcium for enamel mineralization [7].

A number of genes and signaling pathways have been proposed to regulate the cell fate of dental epithelia [3]. Notch signaling controls the differentiation of dental epithelial SI cells [8], and it is also well recognized as a regulator of cell fate in other tissues [9]. The activation of Notch canonical signaling requires the proteolytic cleavage of Notch in the membrane, releasing c-Notch, which enters the nucleus and induces the transcription of target genes such as Hes1 [9]. However, it remains unclear how DE-SCs specify the cell fate of dental epithelia.

Mediator is implicated in maintaining the cell fate of embryonic stem cells (ESC). Mediator specifically regulates four key transcription factors through the formation of the super-enhancer [10], and it is not a general coactivator as previously predicted. Reduced levels of Mediator subunits induce ESC differentiation [11]. In addition, Mediator may regulate cell fate of somatic cells [10], [11]. We demonstrated that the deletion of one of the subunits of Mediator, Mediator 1 (Med1 and also called DRIP205) [12], [13], [14] modulates keratinocyte differentiation *in vitro* [15], [16]. The ablation of Med1 *in vivo*, specifically in the epithelium of mice, activates hair follicles (HF) to accelerate the hair cycle to anagen, whereas the hair shaft was immature leading to mild alopecia in the adult skin [17]. However, the hair and interfollicular epidermis (IFE) are normally developed in early morphogenic stages without Med1. Med1 deletion also disturbed the T cell lineage but not that of B cells [18], [19], and prevented the differentiation of luminal cells but not basal cells in the mammary gland [20], [21]. These results suggest that Med1 controls these cell lineages through adult stem cells residing in these regenerative tissues. In fact, a role for Med1 in skin stem cells was briefly reported [22].

Skin epithelia are also derived from the ectoderm and differentiated into the IFE and HF during the embryonic developmental process. After birth, adult stem cells residing in the basal layer of the epidermis and in the HF bulge (HF-SC) continuously regenerate the epidermis and HF, respectively. In the epidermis, when epidermal stem cells leave the basal layer, they differentiate and express proteins such as keratin 1 (Krt1), Krt10, and loricrin (Lor). HF-SCs originate from the bulge and differentiate as the cells move from the outer root sheath to the inner root sheath. Hair keratins such as Krt31/Ha1 and Krt71 are produced to comprise the hair shaft. Several signaling pathways and hair lineage transcription factors including Gata3, Ovol1, Grhl1, and AP-1/AP-2 (Tcfap) are induced. Epigenetic analysis has revealed HF-SC signature genes that play a role in adult stem cells in HF [23]. Calcium plays an important role in promoting epidermal [24] and hair differentiation [25].

In the current study we show that deletion of Med1 *in vivo* postnatally generates ectopic hairs in the incisors. Med1 ablation causes a cell fate switch by reducing Notch1 signaling and inducing a calcium driven epidermal fate.

## Results

### Med1 Conditional Null Mice Form Hairs in the Incisors while Disrupting Enamel Formation

**Figure 1:**
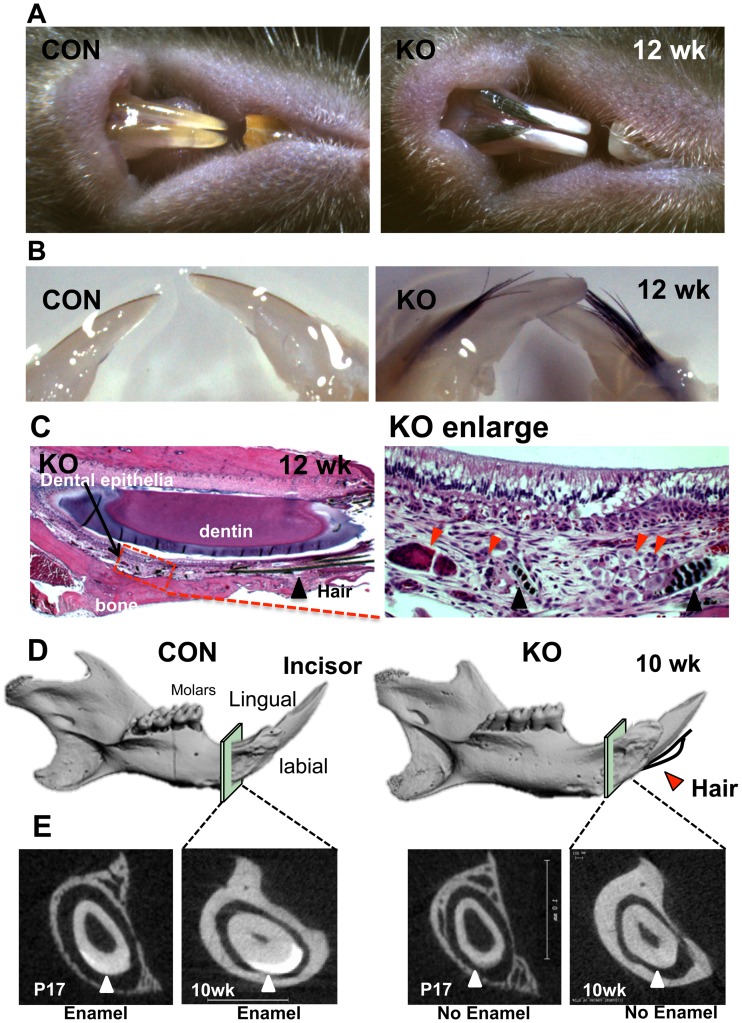
Deletion of Med1 generated hairs on the labial side of incisors while disrupting enamel formation. (A, B) Hairs were generated from the labial side of chalky-colored incisors lacking enamel in Med1 KO mice (12 wk) (A) and in dissected jaws (12 wk) (B). (C) Hairs (black triangle) were observed in the tissue between the bone and the dental epithelial layer (left panel) in Med1 KO. Hairs originated from abnormal tissues (red triangles) underlying the non-polarized ameloblasts and the papillary layer (enlarged image in right panel) (12 wk). (D, E) Micro CT analysis shows the enamel hypoplasia in the Med1 KO. The 3D-reconstructed µCT images showed the structure of the mandible in CON and KO mice (10 wk) and the location of hairs (red triangle) (D). Enlarged sections of the µCT images (squares) are shown (E). The high density mineralized layer (enamel) was present on the labial side of incisors in CON incisors but absent in Med1 KO (triangles) (10 wk) with changes starting at P17. Hair was observed consistently in over 20 litters of Med1 KO mice. The entire mandibles of two mice (CON and KO) from 2 litters (10 and 12 wk) were scanned using µCT, and reproducibility was confirmed. A mineralized layer was never detected in Med1 KO incisors.

Previously, we generated the conditional mouse model, in which Med1 is specifically removed from the epithelium by utilizing floxed (exon 8–10) Med1 mice [26] and the Cre-loxP strategy [17]. Homozygous floxed Med1 mice with the keratin 14 (Krt14) driven Cre transgene (KO) were compared to control littermates that had floxed Med1 alleles but no Cre (CON) (Figure S1A). Med1 was expressed in the dental epithelia OEE/IEE/SR/SI containing DE-SC in the CL of the incisors of control mice (CON) at postnatal day 1 (P1), but was substantially diminished in the conditional null mice (KO) (Figure S1A, B). In Med1 KO mice, the generation of a few hairs was observed in incisors at 4 weeks (wk) of age (Figure S2 KO triangles), but the number of hairs was increased robustly after 8 wk (Figure 1A, B 12 wk). The hairs were located on the labial side of the incisors, and the incisors were notable for their chalky appearance indicating defects in enamel formation (Figure 1A, B). The hair in the incisors persisted throughout life, but was not found in Med1 KO molars. Micro CT (µCT) analyses confirmed the enamel hypoplasia in the incisors of Med1 KO mice (Figure 1D, E). The radio-opaque mineralized enamel layer of the incisors of CON mice was detectable at P17, and increased at 10 wk but mineralized layer was not observed in Med1 KO incisors at any stages in any sections scanned (Figure 1D, E). Histological analysis revealed that the hairs originated from abnormal cell clusters under papillary layers (Figure 1C red triangles Figure S2). The hairs were generated in the same region in which enamel formation was disrupted and in which the vasculature invaded the papillary layers at the maturation stage (Figure 1C). The nuclear polarization of ameloblasts was disturbed in Med1 KO incisors at both 10 wk (Figure 1C) and 4 wk (Figure S2C).

### Med1 Ablation Prevents Dental Fate and Drives Epidermal Fate

**Figure 2:**
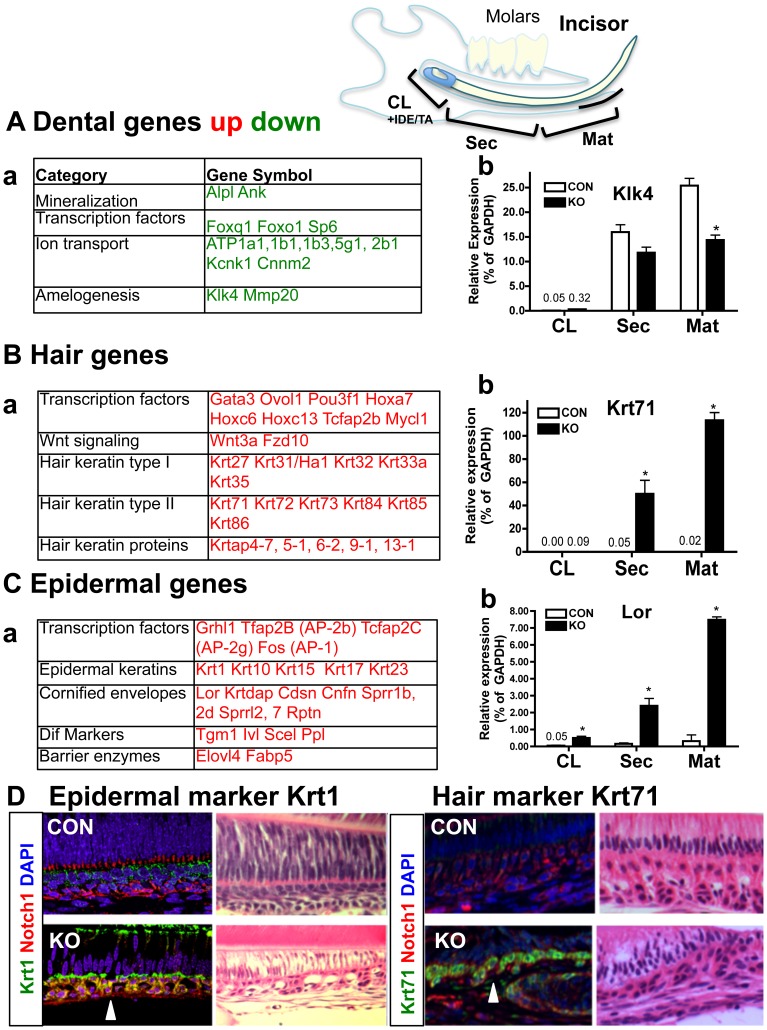
Med1 ablation prevents dental fate but drives epidermal fate. The IPA pathway analysis on 4(A) The expressions of dental genes were decreased in KO at the Mat stage. Instead, hair genes (B) and epidermal genes (C) increased in KO at the Sec and Mat stages. The full list of altered genes with fold changes and detailed information is shown in Tables S1, 2 and 3. Green letters indicate down-regulation and red letters indicate up-regulation. The mRNA levels of representative genes for dental (A, b Klk4), hair (B, b Krt71), and epidermal (C, b Lor) are confirmed by QPCR. The average and SD of relative expressions (% of GAPDH) in CL, Sec and Mat at 4 wk are shown. The numbers show the value of the relative expression, which are too low to represent using bars. The statistically significant increases/decreases in Med1 KO (closed bars) compared to KO (open bars) at each stage are shown by asterisks (n = 3, *p*<0.05). (D) Immuno-staining of epidermal keratin Krt1 (left) and hair keratin Krt71 (right) (Krt1 or Krt71 green, Notch1 red, DAPI blue) in the Med1 KO and CON at 4 wk. Equivalent sections stained by HE are presented on the right side of each image.

We then investigated how Med1 deletion changes epithelial cell fate. First, we examined gene expression profiles in Med1 KO and CON using expression arrays. Tissues were micro-dissected from the mandibles of KO and CON mice at three different locations; 1) CL (+IDE/TA), 2) secretory (Sec) stage, and 3) maturation (Mat) stage (Figure 2 diagram) at 4 wk when hair growth in the incisors was first observed. RNA was purified and gene expression profiles were analyzed using an Illumina beads-based gene array including 25,600 annotated transcripts and 19,100 genes (data for CL, Sec, Mat tissues in Med1 KO and CON at 4 wk are available in GEO database GSE50503). The array data were analyzed using IPA software. These analyses revealed down regulation of dental genes (Figure 2A, Table S1) and the induction of hair and epidermal genes (Figure 2B, C, Table S2, S3). Dental epithelia differentiate as they move from the CL to the Sec and Mat stages. However, Med1 deletion decreased the expression of dental genes: alkaline phosphatase (Alpl/akp2), Na and K transporting ATPase [7] (SI differentiation), forkhead box O1 (Foxo1) (enamel mineralization) [27], and kallikrein-related peptidase 4 (Klk4) [28] and matrix metallopeptidase 20 (Mmp20) (enamel maturation) (Figure 2A, Table S1). However, enamel matrix proteins such as ameloblastin and amelogenin did not change. Transcription factors that direct hair lineage, such as Ovol1 and Hoxc13, were induced (Figure 2B). Similarly, factors that induce epidermal differentiation, such as AP-1/2 (Fos, Tfap2B), increased in the KO dental epithelium (Figure 2C). Genes involved in skin melanogenesis such as Tryp6 were also induced. A full set of genes for proteins that comprise the hair follicles and epidermis during their differentiation were also induced (Figure 2B, C, Tables S2, S3). The array data were confirmed by QPCR analysis (Figure 2 bar graphs). Dental gene Klk4 decreased in Med1 KO compared to CON at the Mat stage (Figure 2A b). The hair gene Krt71 was not detected in the CON but increased at the Sec/Mat stages in Med1 KO (Figure 2B b). In addition, the epidermal gene Lor also increased in the Sec/Mat stages in Med1 KO (Figure 2C b). Immuno-staining showed that epidermal keratin Krt1 and hair keratin Krt71 were expressed in Med1 KO dental epithelia, although they were not detected in CON (Figure 2D). These results demonstrated that the Med1 deficient dental epithelium differentiated into epidermal and hair expressing epithelium probably by following the gene expression program of skin epithelia.

### Med1 Ablation Results in Defects in Niche Architectures

**Figure 3:**
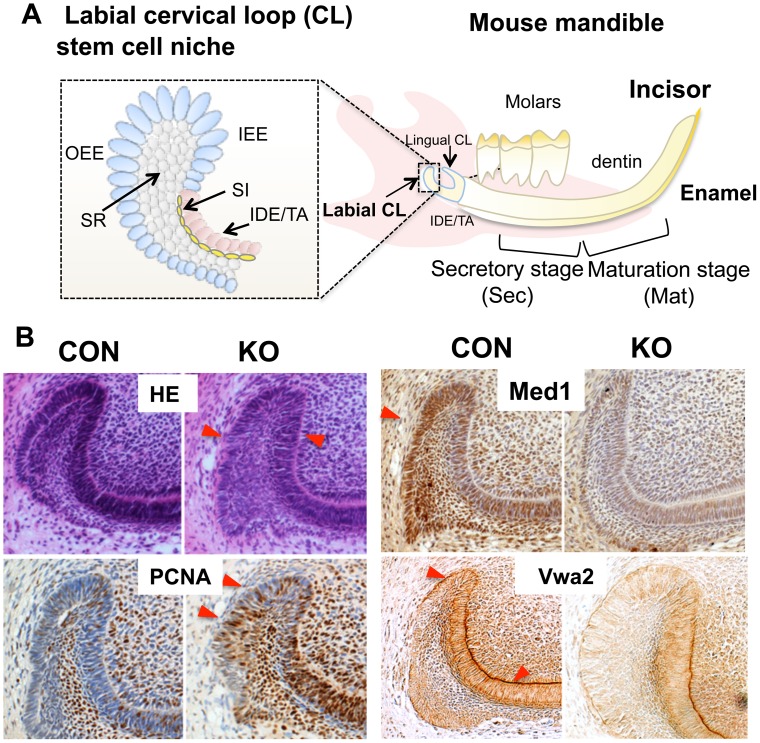
Med1 ablation resulted in defects in the DE-SC niche. (A) A diagram to show the location of the labial CL in mouse mandible, where DE-SCs reside. The CL is composed of OEE, IEE, SI, and SR (left). (B) Histological analyses of CL in Med1 KO and CON at 4 wk. Med1 is abundantly expressed in OEE/IEE/SR in the CL but diminished in the KO (Med1, brown signals with blue counterstaining). Histological staining shows the morphological alterations of the CL in KO (HE, red triangles). Cell proliferation is increased in OEE/IEE and IDE equivalent to transient amplifying cells (TA) in KO (PCNA staining, brown signals with blue counterstaining). Vwa2 expression in the basement membrane of OEE and IEE decreased in Med1 KO (Vwa2 brown staining with blue counterstaining). The results were reproduced in two independent experiments using two Med1 KO mice from different litters.

We then explored the mechanism by which Med1 ablation changes the cell fate from dental epithelia to skin epithelia. The cell fate of dental epithelia is controlled by dental epithelial stem cells (DE-SC) that reside in a stem cell niche called the cervical loop (CL) in incisors (Figure 3A). Previous studies demonstrated that slow-dividing DE-SC are located in the region of OEE/IEE/SR cells in the labial CL and are capable of differentiating into dental epithelial lineages [1], [29]. Med1 was abundantly expressed there in CON CL but was absent in Med1 KO CL (Figure 3B Med1). It has been shown that functional defects in DE-SCs cause morphological changes in the labial CL [29], [30]. Therefore, the architecture of the stem cell niche was analyzed in the CL region histologically. Med1 deletion did not affect the morphology of the newborn CL (data not shown), but changes were observed at 4 wk, when hair growth in the dental epithelia was first observed. Serial sectioning of the CL shows that the bend in the CL was less pronounced, the SR was expanded, and the OEE/IEE alignment was disturbed in KO compared with CON (Figure 3B HE; serial sections in Figure S3). The cell proliferation of OEE/IEE and IDE equivalent to transient amplifying cells (TA) increased in KO incisors (Figure 3B, PCNA), implicating that DE-SCs were activated. In addition, the expression of extracellular matrix proteins (EMC) decreased. Von Willebrand factor A domain 2 (Vwa2) was expressed at the basement membrane in OEE/IEE and TA but decreased in Med1 KO (Figure 3B Vwa2). Vwa2 was examined because its expression decreased the most in the Med1 KO CL compared to CON in the array analyses shown later (GSE50501). The numbers of other ECM proteins also decreased in the KO. Because Vwa2 is present in hair follicle stem cells [23] and mediates cell adhesion through its RGD domain [31], Med1 deletion may potentially affect the cell-ECM adhesion of DE-SCs and their progenitors.

### Med1 Ablation Extends Sox2 and DE-SC Signature Expression into the Presumptive Differentiation Zone

**Figure 4:**
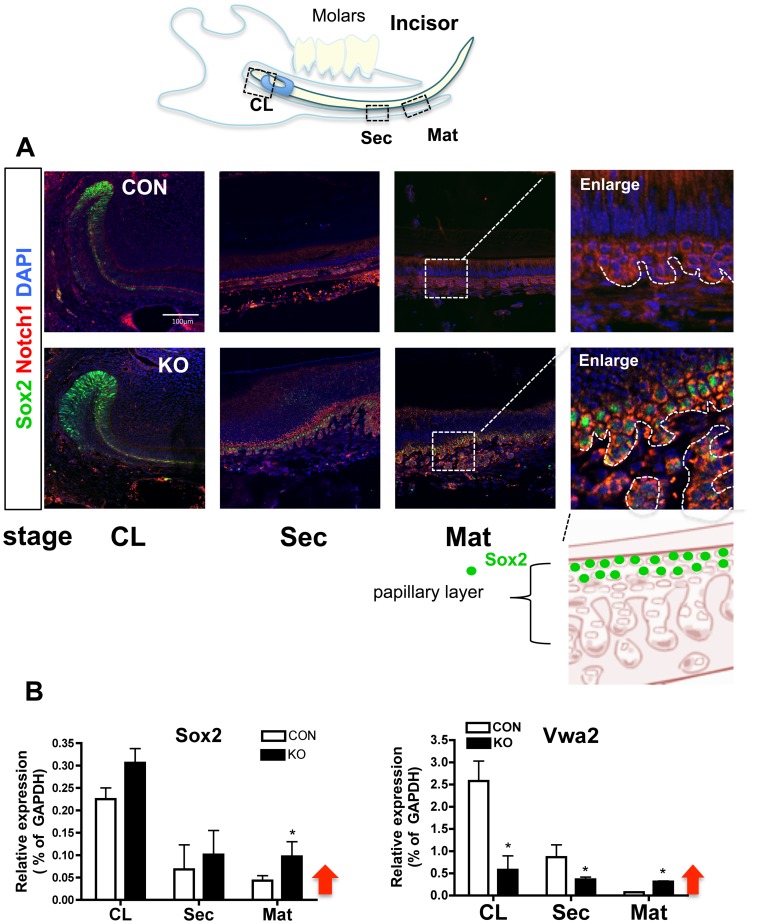
Med1 ablation extended Sox2 and stem cell signatures. (A) Stem cell marker Sox2 expression in dental epithelia at three different stages; the CL (left panels), the Sec (second left), and the Mat stage (third left) in Med1 KO and CON at 4 wk (green Sox2, red Notch1, blue DAPI). The location of the 3 stages is shown in the upper diagram. Enlarged images of the boxed area of the Mat stage are also shown (far right panels), and papillary structures are indicated by dotted lines. The lower diagram shows the location of Sox2 (green) in the papillary layer. (B) The mRNA expression of Sox2 and Vwa2 at three stages (CL, Sec, and Mat) in KO (closed bars) compared to CON (open bars) at 4 wk. The SD of relative expression (% of GAPDH) and the statistical significance (n = 3, * *p*<0.05) are shown. Red arrows indicate their retention in the Mat stage in KO.

Next, DE-SCs were monitored by the expression of the stem cell marker Sox2. Sox2 was enriched in CL but disappeared as dental epithelial cells differentiated into the Sec and the Mat stages in CON incisors (Figure 4A CON). However, Sox2-expressing cells extended into the SI and the papillary layer in the Sec and the Mat stages in the Med1 KO (Figure 4A Mat enlarged image). The locations of Sec and Mat stages are shown in a diagram in Figure 4. The mRNA levels of Sox2 also remained elevated at the Mat stage of KO incisors (Figure 4B Sox2 red arrow). The mRNA expression of the DE-SC signature of Vwa2, showed a similar expression pattern, in which Vwa2 remained higher in KO compared to CON (Figure 4B Vwa2 red arrow). The expression of the HF-SC signature transcription factors that direct cells to hair lineage including Gata3 [32] and Pou3f1/Oct6 [23] were robustly induced at the Mat stage of KO incisors (Table S2 transcription factors). These results demonstrate that Med1 ablation extended Sox2-expressing DE-SCs into the Sec and the Mat zones. Our results suggest that DE-SCs may migrate out to a differentiation zone by increasing proliferation and decreasing adhesion capability. In addition, Med1 ablation induced several transcription factors associated with HF-SC, such as Gata3, Pou3f1/Oct6, Ovol1, Hoxb7, c6, and c13 [23], demonstrating that extended dental stem cells may be converted into HF lineage cells.

### Med1 Ablation Alters DE-SC Cell Fate Through Notch1 and Calcium Signaling

**Figure 5:**
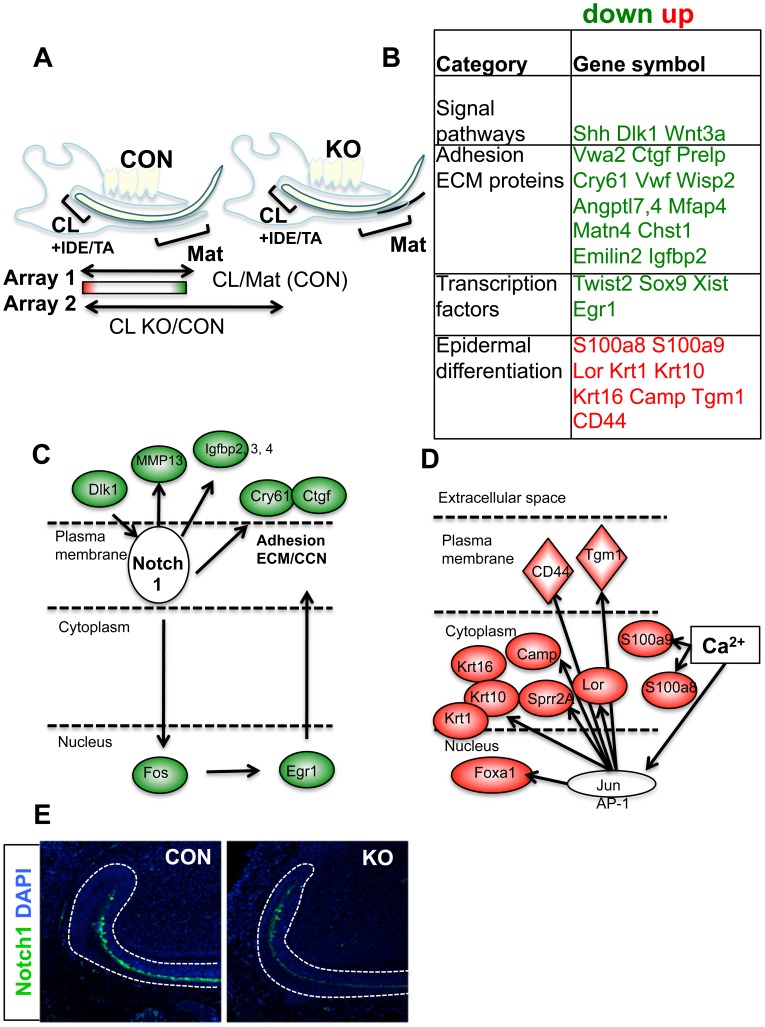
Gene expression profiling predicted changes in Notch and calcium signaling upon Med1 deletion. (A) Strategy of microarray analyses. CL and Mat tissues were dissected from Med1 KO and CON mice at 4 wk and 10 wk. Array 1 calculated fold changes in CL/Mat in control mice revealed genes that are enriched in CL compared to the Mat stage, which are DE-SC signature candidates. Array 2 calculated fold changes in KO/CON at CL shows genes up-or down-regulated in Med1 KO compared with CON. Arrays were performed on RNA samples from Med1 KO and CON. (B) These analyses revealed a list of DE-SC signatures affected in the CL of Med1 KO in comparison with gene pools identified through other stem cells of ESC or HF-SC. Green letters show down-regulation, and the red letters indicate up-regulation. Fold increases and other details are listed in Table S4 (10 wk). (C, D) The IPA pathway analysis predicted that Notch1 (C) and calcium (D) are potential upstream regulators to cause these changes in Med1 KO. The sub-cellular location and the up- and down-regulation of genes are shown by red and green, respectively. (E) The protein expressions of Notch1 at 4 wk are shown in CL of Med1 KO and CON.

We then investigated whether the gene expression profile is altered in the stem cell niche of Med1 KO using expression arrays. The analyses comparing changes in the CL/Mat in the control tissues at 10 wk revealed potential DE-SC signatures (Figure 5A Array 1), that were enriched in the CL compared with the Mat (Table S4 red numbers). We further conducted a second array analysis to identify which genes are affected in the KO CL compared with the CON CL at 10 wk (Figure 5A Array 2) (GSE50501). We also analyzed these changes at 4 wk. We further selected genes essential for stem cell function by comparing them with HF-SC signatures, which were previously identified through the epigenetic status of HF-SC [23]. They were also compared with ESC derived tissue specific lineage genes [23]. These comparisons revealed a short list of DE-SC signature genes, which are conserved in HF-SC or ESC but down-regulated in Med1 KO compared with CON at 10 wk (Figure 5B, Table S4). The stem cell niche transcription factors or lineage determinants, such as Sox9 [33], Twist2 [34] and Egr1, decreased. The signaling component of Shh, which controls the cell fate of dental epithelia, was also down-regulated. In addition, a significant number of extracellular matrix (ECM) proteins including CCN family members were down-regulated affecting cell adhesion as previously shown in Figure 2 (Figure 5B, Table S4 green numbers). On the other hand, epidermal markers were already induced in the CL fraction at 4 wk probably because it contains IDE/TA and the proximal portion of the secretion zone. Pathway analysis (IPA) and our knowledge predicted upstream regulators inducing these changes in CL. Notch1 was an inhibitory regulator at both 4 wk and 10 wk. Notch ligand Dlk1 decreased, and Notch1 regulated matrix proteins such as Ctgf and Cry61 decreased [35] which potentially supports stem cell adhesion and differentiation [36] (Figure 5C). The Notch1 protein was also reduced in the CL of Med1 KO incisors compared with that of CON incisors (Figure 5E). IPA also predicted that calcium is a potential activating regulator to induce epidermal differentiation at 4 wk. Calcium functions through calcium binding proteins, such as S100a8 and a9, and through AP-1 factors, which would induce Lor or Krt1 through their binding to calcium response elements in the promoter of these genes (Figure 5D). These results demonstrate that Med1 regulates fate transcription factors in somatic stem cells. Med1 ablation causes cell fate switch by preventing dental differentiation probably by reduced Notch signaling and facilitating calcium induced epidermal differentiation.

### The Role of Calcium and Notch1 in the Med1 Regulation of Dental Epithelial Differentiation *in vitro*

**Figure 6:**
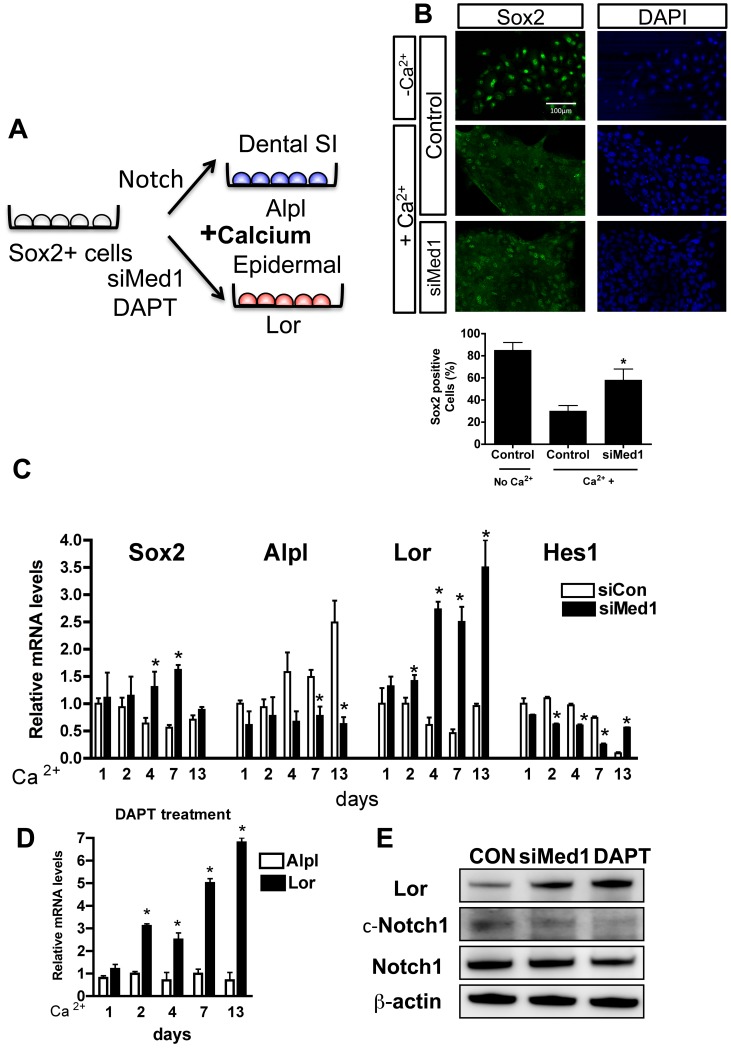
Med1 regulates cell fate through calcium and Notch signaling. (A) Cervical loop derived dental epithelial (CLDE) cells were established, and Med1 was silenced by siRNA (siMed1). (B) Sox2 was present in CLDE cells that were maintained in low calcium (upper panels), was decreased in high calcium (1.5 mM) (middle panels) (4 days culture), but sustained in siMed1 cells in high calcium (lower panels) (Sox2 green, DAPI blue). The numbers of Sox2 positive cells were counted (bar graph). (C) Control (open bars) and siMed1-treated cells (closed bars) were maintained for 1–13 days in 1.5 mM calcium, and the mRNA expression of Alpl (blue in A), Lor (red in A), Sox2, and Hes1 were measured. Data are shown as the mean±SD of triplicate measurements. The statistically significant increases or decreases in siMed1 compared with sicontrol at each stage are shown by asterisks (*p*<0.05). (D) Notch inhibitor DAPT suppressed the induction of Alpl, but induced the expressions of Lor (* *p*<0.05) when cells were maintained in a high calcium condition. The control mRNA level at Day 1 without DAPT for Alpl or Lor was set as 1. (E) The protein levels of Lor, cleaved Notch (c-Notch), total Notch, and the loading control β-actin (4 days in high calcium) are shown by the Western blot analysis. The results were reproduced in two different batches of CLDE cells.

**Figure 7:**
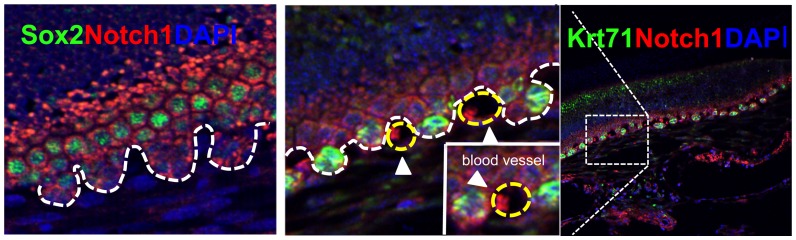
Hair differentiation was induced adjacent to the blood vessels in which calcium is supplied. (A) Hair keratin Krt71 was detected in the papillary layer adjacent to the blood vessels (right panel). Enlarged images are shown in the middle panel (Krt71 green, Notch1 red, DAPI blue). Yellow dotted circles and white arrows show the blood vessels in which erythrocytes are detected (insert). Sox2 was expressed in basal cells in the papillary layer (left panel, Sox2 green, Notch1 red, DAPI blue).

CL-derived dental epithelial (CLDE) cells were established to examine the impact of Med1 deletion on cell fate *in vitro*. The roles of Notch1 and calcium on CLDE cell differentiation were also examined (Figure 6A). Tissues from the CL region of mouse embryonic incisors were dissected, and epithelial cells were cultured at a low density until self-renewing colonies were developed and naturally immortalized. Most of the CLDE cells expressed the stem cell marker Sox2 when they were maintained in a serum free medium with low calcium (Figure 6B -Ca^2+^). The number of Sox2-positive cells decreased in the presence of high calcium (1.5 mM) (Figure 6B +Ca^2+^ for 4 days) indicating differentiation. However, when endogenous Med1 expression was silenced by siRNA (typical mRNA blocking efficiency was 82.5±2.4%), the number of Sox2-positive cells remained high even in a high calcium medium (Figure 6B). The CLDE cells were maintained in the high calcium medium for 1–13 days to examine whether calcium affected Sox2 and cell differentiation. The Sox2 expression decreased with high calcium, while Med1 silencing abrogated this Sox2 reduction until 7 days in the culture, indicating that Med1 deletion prolonged stem cell potential. Dental and epidermal differentiation was also monitored by analyses of the mRNA expression levels of the dental SI marker Alpl and the epidermal marker Lor, respectively (Figure 6C). When Med1 was silenced, calcium-induced epidermal differentiation was shown by the time-dependent increase in Lor mRNA levels, while dental SI differentiation (Alpl) was not induced (Figure 6C, E). Next, we examined the role of Notch signaling in Med1 regulated dental epithelial differentiation and cell fate determination in the CLDE cells. When Med1 was silenced, the expression of the Notch target gene Hes1 decreased for 7 days compared with the control cells (Figure 6C Hes1). This effect was associated with a lower level of cleaved Notch (c-Notch) (4 day culture), although the full-length Notch1 level was not affected (Figure 6E). When cells were treated with DAPT, a γ-secretase inhibitor, to suppress Notch1 cleavage (Figure 6E DAPT c-Notch1), the induction of Alpl was suppressed, whereas expression of Lor was induced similar to the effect of Med1 silencing (Figure 6D). These results suggest that Med1 is required for the activation of Notch1 target genes, such as Hes1, which is involved in dental epithelial cell fate determination, although calcium is required to drive differentiation of the Sox2-expressing cells to an epidermal fate *in vitro*. We confirmed that hair keratin Krt71 expression is increased in the papillary cells adjacent to blood vessels during the Mat stage of Med1 KO incisors, where calcium is supplied for enamel formation (Figure 7).

**Figure 8:**
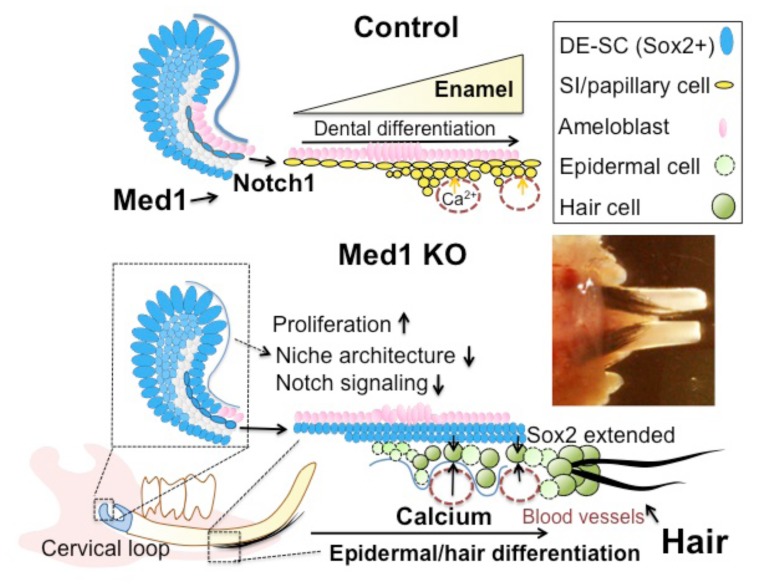
A proposed model in which Med1 ablation alters epithelial cell fate. Med1 regulates Notch signaling by activating Notch1 target genes (upper diagram). Med1 maintains the niche architecture containing Sox2-expressing dental epithelial stem cells (DE-SC) (blue). Enamel is formed as dental epithelia differentiate in control incisors partly due to Notch signaling. In contrast, DE-SCs fail to commit to the dental lineage when Med1 is ablated (lower diagram). Instead, Sox2-expressing cells (blue) extend into the differentiating zones. Med1 deficient cells are exposed to extracellular calcium through the blood vessels (red-dotted circles) adjacent to the papillary layers (yellow). Med1 deficient cells are differentiated into epidermal cells (light green) and hair keratinocyte-like cells (green) and produce mature hair shafts (black) in the Med1 KO incisors (inserted picture).

In summary, we show a schematic diagram (Figure 8) in which we propose that Med1 ablation affects the differentiation of the dental epithelium in two locations. In the CL, Med1 deletion prevents the DE-SC commitment to the dental lineage and sustains Sox2 expression. Our results implicate that Med1 ablation reduces Notch signaling, increases proliferation, and prevents the formation of the appropriate stem cell niche architecture to retain Sox2-expressing DE-SCs in CL. Therefore, Sox2-expressing cells extend well into the Sec and the Mat zones, and Med1 deficient cells then establish a new differentiation center. The cell fate switches to the epidermal lineage by aberrant Notch signaling and by access to extracellular calcium from the vasculature in the papillary layer that facilitates epidermal and hair follicle differentiation without Med1.

## Discussion

### Med1 Regulates the Proliferation and Differentiation of Adult Stem Cells to Govern Dental Epithelial Cell Fate

Our studies strongly suggest that incisor-specific adult stem cells and their regeneration process are required for ectopic hair formation in Med1 KO mice because 1) hairs are formed only in the incisors but not in the molars, 2) hairs are generated at the expense of enamel, and 3) hairs are post-natally generated and continuously regenerated even after hairs are depilated (data not shown). Unlike humans, mouse incisors continuously grow throughout their life because of the adult stem cells residing in the CL. The stem cell maintenance and cell fate decision are supported by the cellular microenvironment (niche) [37]. Stem cells may exchange signals with cells or ECM to maintain their multi-potency to differentiate. Our array analysis revealed that Med1 ablation resulted in major changes in niche ECM proteins. Niche ECM proteins in DE-SCs, such as Vwa2, collagen Col18a1, Ctgf, Slit2 [38] and CCN family members, may mediate cell adhesion and control the migration and the proliferation of DE-SCs [39]. Med1 ablation led to increased proliferation of OEE cells, and Sox2 and other stem cell signature genes continued to be expressed in the differentiating zone consistent with the aberrant differentiation program. Our previous study of Med1 ablation in the epidermal keratinocytes of the skin also demonstrates that an aberrant differentiation in the hair follicle cycle is accelerated into anagen (growth phases of the cycle), perhaps due to the activation of bulge HF-SC, but mature hair follicles capable of producing hair shafts do not develop [17]. *In vitro* Med1 silencing increased proliferation but decreased the formation of the E-cadherin/β-catenin complex that is critical for both cell adhesion and keratinocyte differentiation [15]. When the niche specific ECM protein Col17a1 is deleted from bulge HF-SCs [40], the hair follicle cycle is shifted to anagen with a skin phenotype similar to that of Med1 null mice [17]. Thus, the disruption of DE-SC differentiation by Med1 deletion may be consistent with the disruption of SC differentiation in the hair follicle of the skin, although Med1 ablation in DE-SCs leads to the production of the hair shaft in the wrong place, but the loss of hair shaft production in the right place (i.e. the skin). Calcium may potentiate this process in the incisors. The hair shafts appear in the region in which blood vessels are in close proximity to the papillary zone of the dental epithelium that under normal circumstances provides for the transport of calcium through the overlying SI and ameloblasts into the enamel matrix. Calcium is a well-known stimulator of keratinocyte differentiation [24]. When Sox2-expressing dental epithelial cells are exposed to calcium, their differentiation toward the hair phenotype can be stimulated. Med1 was not required for epidermal differentiation, which resembles the processes in the Med1 KO skin, in which the hair and the epidermis are developed normally in the embryonic and the morphogenic stages (P1–17) [17]. Calcium-induced epidermal differentiation may be driven by AP-1 factors, which may facilitate their transcription without Med1. These results suggest that dental epithelia employ Med1 independent hair and epidermal morphogenesis that occurs in early stages of skin development. Mediator may form the complex without one subunit of Med1, which still functions even though the conformation or the interface may be altered.

### Med1 Controls Cell Fate Through Calcium and Notch1 Signaling

Our pathway analysis of the array data predicted that these changes induced by Med1 ablation could be due to the disruption of Notch signaling because Notch regulates the expression of the matrix CCN family members either directly and/or indirectly [35]. Our *in vitro* studies of Sox2-expressing dental epithelial cells showed that Med1 ablation resulted in defects in the dental epithelial SI lineage through Notch signaling. The Notch inhibitor DAPT blocked the expression of AlpI, a marker of SI cell differentiation, and shifted differentiation toward an epidermal phenotype as observed with Med1 silencing. Previous studies have also shown the role of Notch1 signaling in dental epithelia [41], [42]. Moreover, earlier studies show the recruitment of Med1 to the Hes1 promoter along with Notch1 (Med1 is also called Med220) [43]. Coactivators and corepressors of Notch signaling are known to regulate cell fate [9]. In Drosophila, the manipulation of Notch coregulators stimulates an epidermal phenotype with extra hair-like bristles at the expense of sensory neurons [44]. Thus in the absence of Med1, the disruption of Notch signaling may account for the change in dental epithelial differentiation toward an epidermal fate coordinated with calcium available for enamel formation in dental tissues. Although ameloblast differentiation and enamel matrix secretion were relatively normal, ameloblast maturation and mineralization decreased in Med1 KO incisors, which may explain enamel hypoplasia.

### Med1 KO Model Shows Hair Regeneration Potential

Because both enamel and epidermal lineages are derived from the ectoderm, they likely share some signaling pathways involved with differentiation. Epidermal lineage may be the default fate for DE-SCs, such that Med1 deletion or other stimuli in dental cells may induce an epidermal fate. In fact, hair was accidentally regenerated during experiments undertaken to generate tooth germs (personal communication) in which ES-derived dental epithelia and dental mesenchyme were transplanted under the renal capsules [45]. Because hair is difficult to regenerate, our study demonstrates the potential that human hair could be regenerated by other epithelial cells.

In summary, we propose a model in which Med1 ablation affects the stem cell function at two locations. Med1 may be a master regulator not only for ESC but also for adult stem cells to determine cell fate. Med1 regulation through a Notch signaling partnership with calcium induces a cell fate switch. Our study shows the potential to develop a new hair regeneration therapy to treat diseases such as alopecia or ectoderm dysplasia.

## Materials and Methods

### Krt14-driven Med1 KO Mice

Conditional Med1 KO mice were generated as described in Figure S1 [17]. Briefly, floxed (exon 8–10) Med1 mice [26] (C57/BL6 background) were mated with transgenic mice expressing *Cre* recombinase under the control of the keratin 14 (Krt14) promoter (The Jackson Laboratory, C57/BL6). Genotyping was performed by PCR as previously described [17]. All of the experiments were approved by the Institutional Animal Care and Ethics Committee at the San Francisco Department of Veterans Affairs Medical Center.

### Microcomputed Tomography (µCT) Analyses

To assess tooth mineral content and structure, the mandibles of Med1 KO and littermate controls (CON) at P17 and 10 wk were scanned using microcomputed tomography (µCT). The mandibles were fixed in 10% phosphate-buffered formaldehyde (PBF) for 24 hours, stored in 70% ethanol, and scanned using a Scanco vivaCT 40 scanner (Scanco Medical AG, Basserdorf, Switzerland) with a 10.5 µm voxel size and a 55-kV X-ray energy. Serial cross-sectional scans were performed on the whole mandible. A threshold of 1400 µg hydroxyapatite (HA)/mm^3^ was applied to segment mineralized enamel from bone and soft tissue, and 3D reconstructions were created using the manufacturer’s software.

### Surgical Dissection of Dental Tissues

Hair-producing dental tissues and equivalent control tissues were dissected from the mandible of Med1 KO and control littermate mice. The dental tissues between bone and dentin were removed under a dissecting microscope. At adult stages of 10 wk or later, relatively transparent dental tissues containing slightly pink or brown colored patches (melanin-producing) generated abundant hairs in Med1 KO mice in the location where control mice produced a yellow-colored enamel organ (Mat stage). At 4 wk, hairs were not observed under the dissecting microscope, but equivalent tissues to the adult stage were dissected from Med1 KO and CON mice. In addition, the CL and the tissue from the Sec stage located between the CL and the hair/enamel generating tissues (Mat stage) were also collected. Surgical methods were established to obtain an average variation of 4 samples (each combining tissues from 3 mice and 6 incisors) lower than 20% by GAPDH measurements in the QPCR analysis.

### RNA Preparation From Dental Tissues for Microarray Analysis

Total RNA was prepared using a Pico Pure RNA purification kit (ABI) from dental tissues collected from Med1 KO and CON mice. The total RNA quality was assessed using a Pico Chip on an Agilent 2100 Bioanalyzer (Agilent Technologies).

### Microarray Analysis

Gene expression profiles were analyzed using an lllumina beads chip based gene array (Mouse Ref 8 v 2.0) including 25,600 annotated transcripts and 19,100 genes. Sample preparation, labeling and array hybridizations were performed according to standard protocols from the UCLA Neuroscience Genomics Core Facilities. The data were normalized in the Genome Studio (Illumina). First, we confirmed the quality of the hybridization, the microarray data extraction, and the initial analysis by preliminary experiments by using dental tissues collected from a different number of mice at different stages (3–10 wk). Then, we evaluated differentially expressed genes at 4 wk and 10 wk by using Med1 KO and littermate controls (n = 3). The fold changes (log) in gene expression of Med1 KO over control were calculated (average intensity, *p*-values, and standard deviation). The data were then imported into the Ingenuity IPA software (Ingenuity) via data transformation by setting the fold change over and under 1.5 and the *p-*value less than 0.005. Several analyses performed to predict causal network, pathway, and upstream regulators were conducted using IPA software. The upstream molecules including transcription factors and small molecules were listed, potentially responsible for the observed changes in gene expression. The statistical significance of upstream regulators was evaluated by the z-score and the *p-*values calculated through the algorithm installed in IPA software. The array data were submitted to a public database (GEO/NCBI/NIH http://www.ncbi.nlm.nih.gov/geo). Data at 4 wk (CL, Sec, Mat in KO and CON) are available using accession number GSE50503, and data at 10 wk (CL in KO and CON) can be accessed in GSE50501 under the super-series GSE50504.

### Real Time PCR to Confirm Array Results

A real time PCR (QPCR) analysis was conducted to validate the microarray results using primers to a subset of genes from the microarray. Total RNA was isolated from dental tissues as described above. cDNA synthesis was performed using a TaqMan Reverse Transcription Reagents kit using random hexamer that was provided (Applied Biosystems, N808-0127, Foster City, CA). Real-time quantitative PCR was performed using Power SYBR Green (Applied Biosystems, 1102115) with the 7300 or 7500 Real Time PCR system (Applied Biosystems). Relative mRNA levels compared to the control gene GAPDH were determined using the ΔΔCt method. Primers for the QPCR analysis were designed using the Primer Express Software (Applied Biosystem) to span the exon and intron boundaries, or they were derived from the Primer Bank. Primer sets were verified by drawing dissociation curves. The primer sequences used for dental tissues are available upon request. Analysis was conducted using three independent litters of Med1 KO mice. Averages and standard deviations of relative mRNA expression are calculated. Statistical significance was evaluated by calculating the *p-*values using the Student’s t-test.

### Cervical Loop Derived Dental Epithelial (CLDE) Cell Culture

Tissues from the CL region of the mandible incisors of E15.5 were dissected and digested with type I collagenase (246 U/ml) (Gibco, 17100) and Dispase (1.17 U/ml) (Gibco, 17105). The cells were then cultured in a keratinocyte serum-free medium (K-SFM) (GIBCO, Life technologies, 17005-042) supplemented with EGF and BPE. The CLDE cell line was established through a long culture at a low density until self-renewing colonies were developed without mesenchymal feeder cells. After several passages, epithelial colonies developed. The fibroblasts were not able to survive in the K-SFM medium.

### Reproducibility and Statistical Analysis

All of the experiments using Med1 KO mice were repeated with two or three litters, and reproducibility was confirmed. The experiments using cultured dental epithelia were repeated using two independent batches and showed representative data. The statistical significance was calculated using the two-tailed unpaired Student’s t-test available in Microsoft Excel software (Microsoft Corporation, Redmond, Wash., USA). Differences with *p-*values of less than 0.05 were considered statistically significant and indicated by asterisks.

### Histological Analysis

Mouse mandibles were dissected from Med1 KO and littermate control (CON) mice. They were fixed in 4% paraformaldehyde at 4**°**C and decalcified in 0.5 M EDTA for two weeks. They were dehydrated and embedded in paraffin wax. The whole mandible was serially sectioned at 7 mm (sagittal sections) containing both the CL and enamel organ. They were stained with hematoxylin and eosin (HE).

### Immunostaining

Paraffin embedded jaw sections were treated with antigen unmasking solutions (Vector lab Citrate-based, H-3300). They were blocked by an avidin/biotin blocking kit (Vector lab, SP-2001), and incubated with primary antibodies against Med1 (TRAP220, Santa Cruz), Vwa2 (Santa Cruz Biotech 1∶1000), and loricrin (Covance 1∶1000). They were then developed using DAB staining solution (Vector Lab, SK-4100) and a Vectastain Elite ABC kit (Vector Lab) until brown.

### Proliferation

PCNA-positive cells were detected using a PCNA staining kit (93-1143, Invitrogen) according to the manufacturer’s protocol.

### Immunofluorescence

Paraffin-embedded mandible sections were dehydrated, rehydrated, and then pretreated in 10 mmol/L citrate buffer (pH 6.0, Sigma-Aldrich) for 20 min using a microwave for antigen retrieval. The specimens were blocked by Power Block (BioGenex) and incubated with primary antibodies against Med1 (Santa Cruz, TRAP220), Krt71 (Progen), and loricrin (Covance), Sox2 (Epitomics), Alpl (R&D system), Notch1 (Cell Signaling), and c-Notch1 (Cell Signaling). They were subsequently incubated with species specific secondary antibodies conjugated with fluorescent dyes (Invitrogen Molecular Probes), including Alexa 594 (red) and Alexa 488 (green). They were then counterstained with DAPI. The images were taken with a confocal microscope (LSM510, Carl Zeiss). Pictures were taken under magnification of 200.

### Gene Expression Analysis for CLDE Cells

Total RNA was also isolated from DE cells by using RNeasy kit (Qiagen). cDNA was synthesized by using a random primer with SuperScript III (Invitrogen). PCR amplification was performed with iQ SYBR Green Supermix (Bio-Rad Laboratories) and a CFX96 thermal cycler (Bio-Rad Laboratories). The relative mRNA levels compared to the control gene GAPDH were calculated. The primer sequences used for the analysis are available upon request in addition to those utilized for dental tissue analysis.

### Western Analysis

Cell lysates were prepared by using the CelLytic M cell lysis reagent (Sigma-Aldrich). Protein levels were determined by using Micro-BCA Assay Reagent (Thermo Fisher Scientific) according to the manufacturer’s protocol. Ten micrograms of protein were loaded onto a NuPAGE Bis-Tris Gel. The blot was incubated with antibody against Notch1 (Cell Signaling), c-Notch (Cell Signaling), loricrin (Covance), and β-actin (Abcam), and subsequently with secondary antibody conjugated with HRP. The signals were visualized by an ECL kit (Thermo Scientific).

### siRNA Silencing of Med1 Expression

CLDE cells were incubated in 12 well plates at a density of 2×10^5^ cells/well in the K-SFM medium (Invitrogen). DE cells were transfected with either siRNA for Med1 (Thermo, Darmacon, ON-TARGET Plus L-040964-01) for mouse Med1 or control siRNA (Thermo, D-001810-10) by using Lipofectamine RNAi MAX (Invitrogen) according to the manufacturer’s protocol. The blocking efficiency of Med1 was confirmed by QPCR analysis and Western analysis. DE cells were switched to the K-SFM media containing 1.5 mM calcium to induce their differentiation and maintained for different lengths of time.

## Supporting Information

Figure S1 **Med1 is removed from the dental epithelia in Med1 KO mice.** (A) The gene-targeting strategy to delete the Med1 (exon 8–10) from keratin 14 expressing epithelia by using Cre-loxP system is illustrated. Triangles show the position of QPCR primers used to detect Med1 expression. Bar graph shows that QPCR analysis indicated that the mRNA expression of Med1 was reduced in dental epithelia in KO when compared to littermate control (WT). (B) The cartoon rendering of enamel epithelia in the CL containing dental epithelial stem cells (DE-SC) (upper) and columnar ameloblasts and stratum intermedium (SI) at the secretory stage corresponding C to show a reduction of Med1 protein in Med1 KO compared to control (CON) at P1.
(TIF)

Figure S2 **Dental epithelia generated hairs instead of enamel in Med1 KO incisors at 4 wk**. (A) Med1 KO incisors started to generate a few hairs (triangle) internally from their labial side where the control mice (CON) formed enamel (blue). No hair was observed in their molars. The diagram shows the structures of the teeth and the locations of hair generated in the incisors. (B) Histological assessment of dental tissues in Med1 KO compared to CON. Mandibles were fixed and decalcified, and sagittal sections were stained by HE. Enamel was decalcified and its presence was shown by a large blank space in CON. In contrast, Med1 KO did not have a blank space indicating a lack of enamel. Hair was internally visible in dental tissues (KO triangle). The CL was not included in these sections. (C) High magnification profiles of boxed area of Med1 KO (box c, d) and CON (box a, b) in B. Arrow shows abnormal expansion of dental epithelia of papillary layer (d) compared to CON (b). Triangle shows hair visible in dental tissues (c). Cell proliferation increased in KO (PCNA brown staining with blue counterstaining) (e, f). Red arrow shows dental epithelial area stained by PCNA.
(TIF)

Figure S3 **Med1 deletion resulted in the alteration of the morphology of the CL in Med1 KO incisors.** (A) The morphology of the CL, where dental epithelial stem cells (DE-SC) reside. Serial sections (1–6) of Med1 KO are compared to those of the CON (4 wk). Representative images are shown.
(TIF)

Table S1 **List of genes down-regulated in dental tissues at the Mat stage of Med1 KO (4 wk), which involve in dental epithelial differentiation.** Down-regulated genes (*p*<0.005) are categorized by their function during enamel development.
(PDF)

Table S2 **List of genes up-regulated in dental tissues at the Mat stage of Med1 KO (4 wk), which involve in hair differentiation.** Up-regulated genes (*p*<0.005) are categorized by their function during hair development.
(PDF)

Table S3 **List of genes up-regulated in dental tissues at the Mat stage of Med1 KO (4 wk).** Up-regulated genes (*p*<0.005) are categorized by epidermal differentiation, calcium signaling and cell adhesion involved during development of epidermis.
(PDF)

Table S4 **DE-SC signatures are down-regulated in Med1 KO.** Genes are categorized by comparison to previous epigenetic analysis of ESC or HF-SC. Genes to regulate tissue specific lineage are shown as ESC because they are bivalent or PGc repressed in ESC. Genes specific for HF are shown as HF-SC and TAC. DE-SC marker candidates that are enriched in CL are shown by fold changes of CL/Mat in CON (left column, red numbers *p*<0.005). The significant decrease of DE-SC candidates in Med1 KO compared to CON at 10 wk are highlighted by green numbers (right column, fold changes of KO/CON in CL *p*<0.005). The cellular locations of the genes are also shown beside fold changes.
(PDF)
