# Supplementary figures and images for: Ablation of Coactivator Med1 Switches the Cell Fate of Dental Epithelia to That Generating Hair
Source: PLoS One. 2014 Jun 20;9(6):e99991. doi: 10.1371/journal.pone.0099991 (PMC4065011; doi:10.1371/journal.pone.0099991)

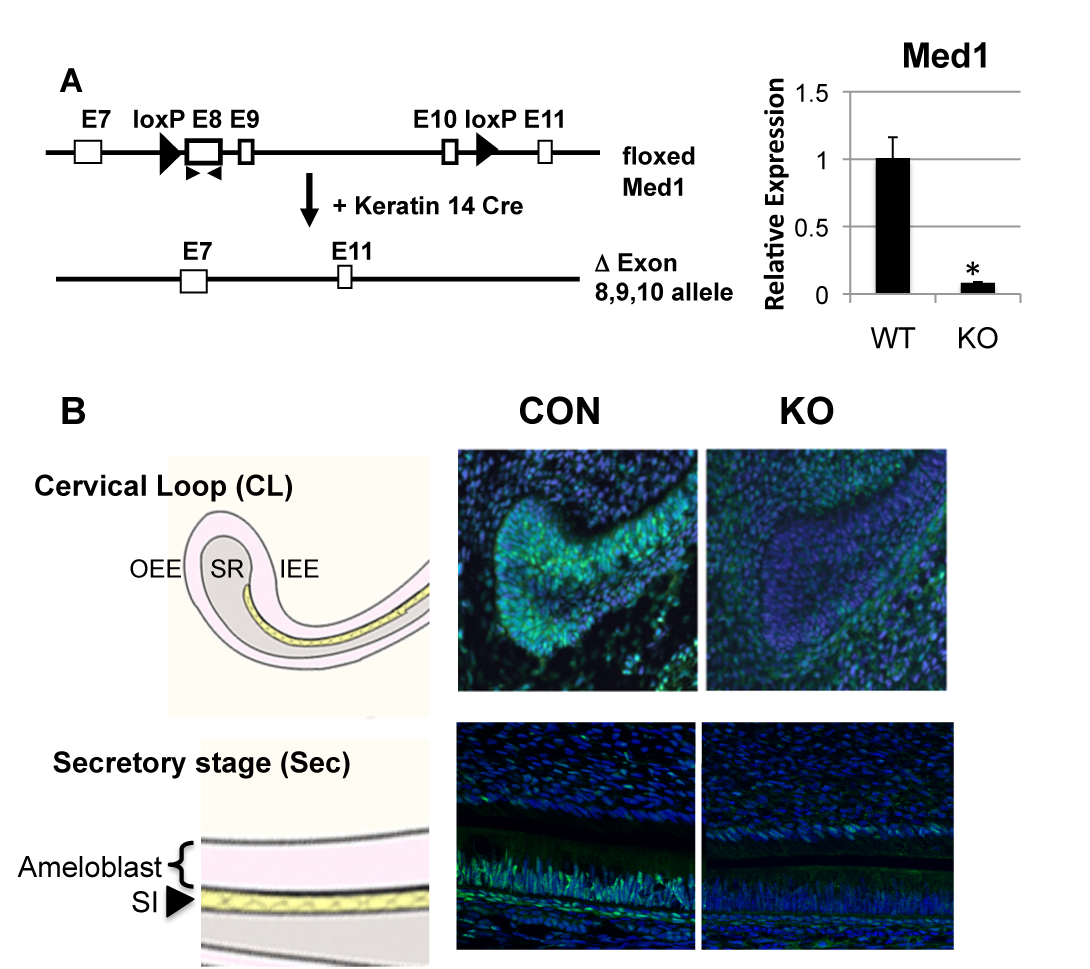

Supplement: Figure S1 — Med1 is removed from the dental epithelia in Med1 KO mice. (A) The gene-targeting strategy to delete the Med1 (exon 8–10) from keratin 14 expressing epithelia by using Cre-loxP system is illustrated. Triangles show the position of QPCR primers used to detect Med1 expression. Bar graph shows that QPCR analysis indicated that the mRNA expression of Med1 was reduced in dental epithelia in KO when compared to littermate control (WT). (B) The cartoon rendering of enamel epithelia in the CL containing dental epithelial stem cells (DE-SC) (upper) and columnar ameloblasts and stratum intermedium (SI) at the secretory stage corresponding C to show a reduction of Med1 protein in Med1 KO compared to control (CON) at P1. (TIF) [file pone.0099991.s001.tif]

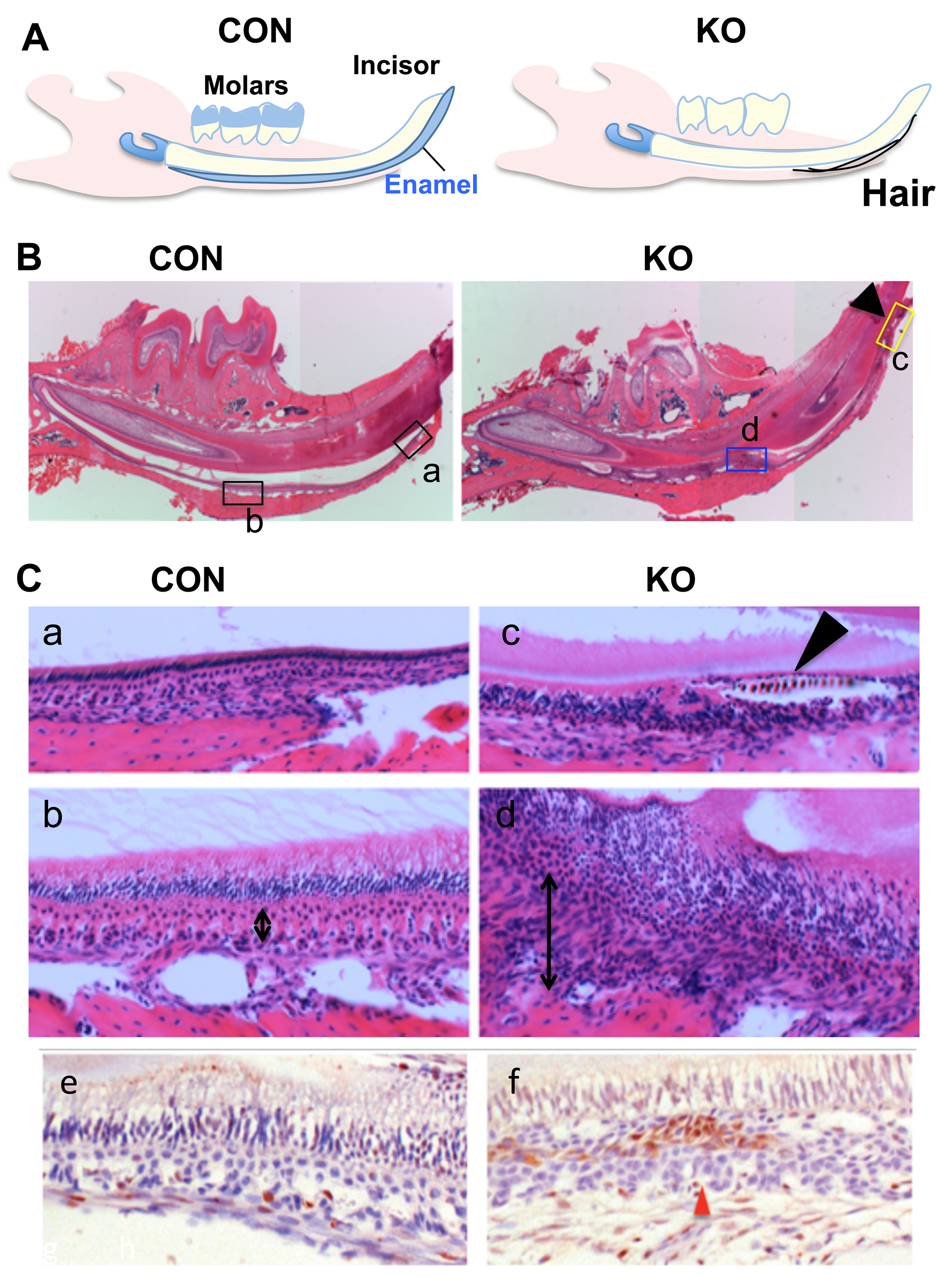

Supplement: Figure S2 — Dental epithelia generated hairs instead of enamel in Med1 KO incisors at 4 wk. (A) Med1 KO incisors started to generate a few hairs (triangle) internally from their labial side where the control mice (CON) formed enamel (blue). No hair was observed in their molars. The diagram shows the structures of the teeth and the locations of hair generated in the incisors. (B) Histological assessment of dental tissues in Med1 KO compared to CON. Mandibles were fixed and decalcified, and sagittal sections were stained by HE. Enamel was decalcified and its presence was shown by a large blank space in CON. In contrast, Med1 KO did not have a blank space indicating a lack of enamel. Hair was internally visible in dental tissues (KO triangle). The CL was not included in these sections. (C) High magnification profiles of boxed area of Med1 KO (box c, d) and CON (box a, b) in B. Arrow shows abnormal expansion of dental epithelia of papillary layer (d) compared to CON (b). Triangle shows hair visible in dental tissues (c). Cell proliferation increased in KO (PCNA brown staining with blue counterstaining) (e, f). Red arrow shows dental epithelial area stained by PCNA. (TIF) [file pone.0099991.s002.tif]

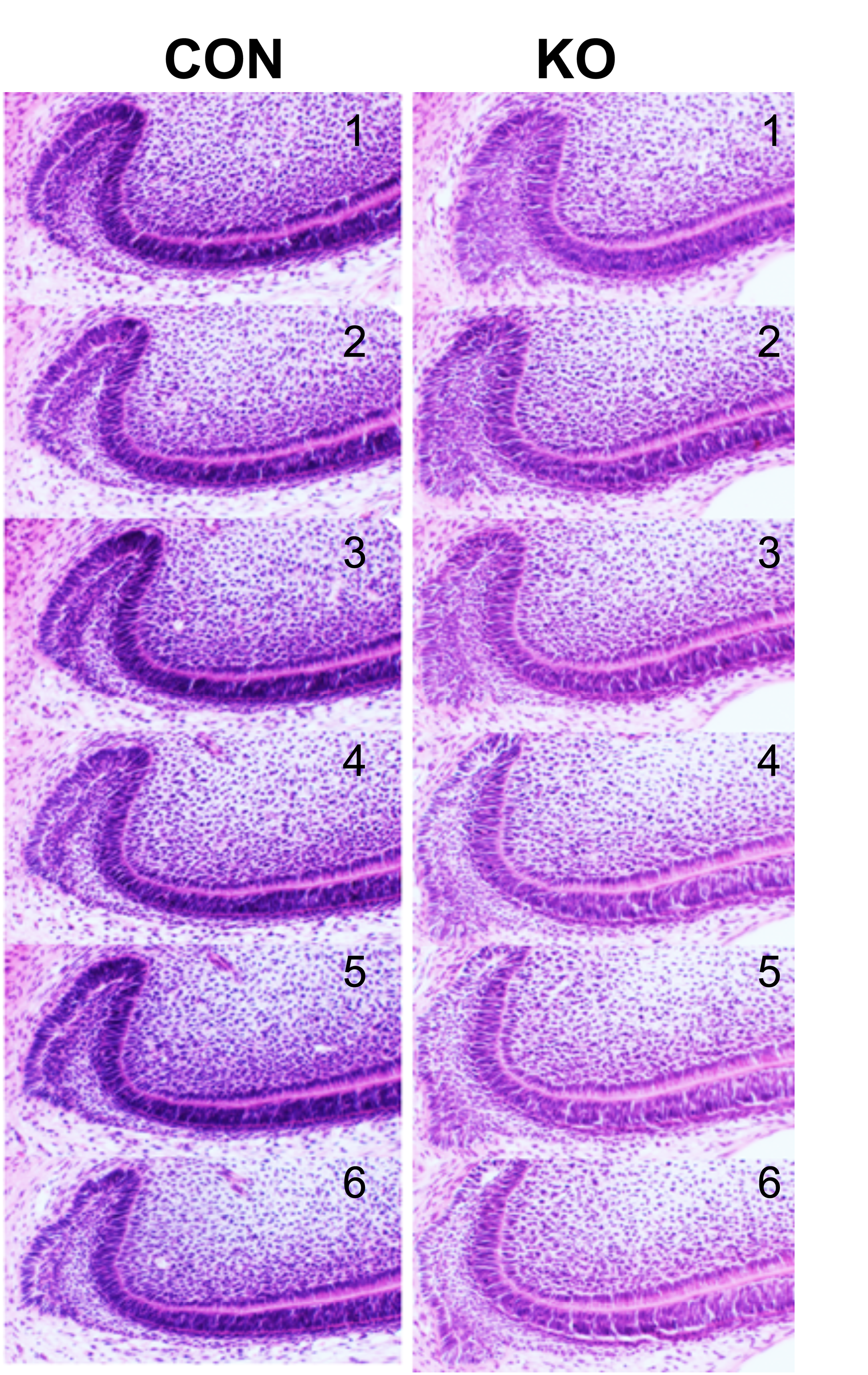

Supplement: Figure S3 — Med1 deletion resulted in the alteration of the morphology of the CL in Med1 KO incisors. (A) The morphology of the CL, where dental epithelial stem cells (DE-SC) reside. Serial sections (1–6) of Med1 KO are compared to those of the CON (4 wk). Representative images are shown. (TIF) [file pone.0099991.s003.tif]
