# Supplementary material for: Ablation of Coactivator Med1 Switches the Cell Fate of Dental Epithelia to That Generating Hair
Source: PLoS One. 2014 Jun 20;9(6):e99991. doi: 10.1371/journal.pone.0099991 (PMC4065011; doi:10.1371/journal.pone.0099991)
Supplement: Table S1 — List of genes down-regulated in dental tissues at the Mat stage of Med1 KO (4 wk), which involve in dental epithelial differentiation. Down-regulated genes (p<0.005) are categorized by their function during enamel development. (PDF) [file pone.0099991.s004.pdf]

# Table S1

| Category | Entrez Gene Name | Symbol | Fold Change | Entrez Gene ID for Mouse |
|---|---|---|---|---|
| **Mineralization** | Ankylosis, Progressive Homolog | Ank | -2.17 | 11732 |
| | Alkaline Phosphatase 2, Liver | Akp2/Alpl | -1.48 | 11647 |
| **Amelogenesis** | Kallikrein related-peptidase 4 | Klk4 | -2.66 | 56640 |
| | matrix metallopeptidase 15 | Mmp20 | -1.62 | 30800 |
| **Ion Transport** | ATPase, Na/K Transporting | Atp1b1 | -5.79 | 11931 |
| | ATP Synthase, H+ Transporting | Atp5g1 | -1.56 | 11951 |
| | ATPase Ca++ Transporting | Atp2b1 | -0.59 | 67972 |
| | Potassium Channel, Subfamily K, Member 1 | Kcnk1 | -4.72 | 16525 |
| | Magnesium Channel | Cnnm2 | -2.11 | 54805 |
| | Solute Carrier Family 40 | Slc40a1 | -4.83 | 53945 |
| | Solute Carrier Family 5 | Slc5a8 | -9.90 | 216225 |
| | Solute Carrier Family 25 | Slc25a1 | -3.22 | 13358 |
| | Solute Carrier Family 39 | Slc39a8 | -2.22 | 67547 |
| | Solute Carrier Family 23 | Slc23a2 | -4.13 | 54338 |
| | Solute Carrier Family 26 | Slc26a4 | -5.14 | 23985 |
| **Tight Junction** | Claudin 12 | Cldn12 | -1.98 | 64945 |
| **Ca transport** | Claudin 1 | Cldn1 | -2.82 | 12737 |
| **Coactivators** | Nuclear Receptor Coactivator 4 | Ncoa4 | -8.09 | 27057 |
| | Cofactor Required for Sp1 Transcriptional Activation | Crsp8/Med27 | -4.05 | 68975 |
| **Others** | Caspase 6 | Casp6 | -1.69 | 12368 |
| | Ubiquitously Expressed Transcript | Uxt | -2.40 | 22294 |
| **Transcription Factors** | Forkhead Box Q1 | Foxq1 | -5.48 | 15220 |
| | Forkhead Box O1 | Foxo1 | -5.22 | 56458 |
| | paired-like Homeodomain Transcription Factor 2 | Pitx2 | -0.48 | 18741 |
| | Isl1 Transcription Factor | Isl1 | -2.05 | 16392 |
| | DNA-Binding Protein Inhibitor | Idb4 | -2.38 | 15904 |
| | General Transcription Factor 3 A | Gtf3a | -2.13 | 66596 |
| | Zinc Finger Protein 185 | Zfp185 | -2.46 | 22673 |
| | Zinc Finger Protein 219 | Zfp219 | -1.79 | 69890 |
| | Zinc Finger Protein 637 | Zfp637 | -1.88 | 232337 |
| | Morc Family Cw-Type Zinc Finger 2 | Zcwcc1 | -2.44 | 74522 |
| | Kruppel-Like Factor 13 | Klf13 | -2.2 | 50794 |
| | Trans-Acting Transcription Factor 6 | Sp6 | -2.32 | 83395 |
| | P53 Inducible Nuclear Protein 2 | Trp53inp2 | -2.11 | 68728 |
