# Supplementary material for: Ablation of Coactivator Med1 Switches the Cell Fate of Dental Epithelia to That Generating Hair
Source: PLoS One. 2014 Jun 20;9(6):e99991. doi: 10.1371/journal.pone.0099991 (PMC4065011; doi:10.1371/journal.pone.0099991)
Supplement: Table S2 — List of genes up-regulated in dental tissues at the Mat stage of Med1 KO (4 wk), which involve in hair differentiation. Up-regulated genes (p<0.005) are categorized by their function during hair development. (PDF) [file pone.0099991.s005.pdf]

Table S2

Category	Entrez Gene Name	Symbol	Fold Change	Entrez Gene ID for Mouse
**Hair Differentiation**	Calcium Binding Differentiation	S100a3	27.74	--
	Peptidyl Arginin Deiminase	Padi1	6.85	--
**Wnt Signaling**	Wingless-related MMTV Integration Site 3A	Wnt3a	2.59	22416
	Frizzled Homolog 10	Fzd10	1.09	93897
	OVO Homolog-like 1	Ovol1	4.64	18426
**Transription foctors**	GATA binding protein 3	GATA3	16.768	14462
	homeobox C13	HOXC13	8.809	15422
	POU class 3 homeobox 1	POU3F1	7.651	18991
	homeobox B7	HOXB7	6.895	15415
	homeobox C6	HOXC6	6.034	15425
**Type I Hair IRS**	Keratin 27	Krt27	7.01	16675
**Type I Hair Keratins**	Keratin 31	Krt31	27.51	16660
	Keratin 32	Krt32	3.50	16670
	Keratin 33	Krt33a	55.88	71888
	Keratin 35	Krt35	16.38	53617
	Keratin 36	Krt36	2.08	16673
**Type II Hair IRS**	Keratin 71	Krt71	18.04	56735
	Keratin 72	Krt72	16.60	--
	Keratin 73	Krt73	21.99	223915
**Type II Hair Keratins**	Keratin 84	Krt84	5.00	16680
	Keratin 85	Krt85	9.98	53622
	Keratin 86	Krt86	41.26	--
**Hair Related**	Keratin Associated Protein 4-7	Krtap4-7	8.02	76444
**Keratin Associated Proteins**	Keratin Associated Protein 4-11	krtap4-11	4.54	665891
	Keratin Associated Protein 5-1	Krtap5-1	2.09	50774
	Keratin Associated Protein 6-2	Krtap6-2	3.9	16701
	Keratin Associated Protein 9-1	Krtap9-1	6.95	--
	Keratin Associated Protein 13-1	Krtap13-1	0.96	69696
	Keratin Associated Protein 16-3	Krtap16-3	10.46	170653
	Keratin Associated Protein 16-4	Krtap16-4	6.98	170654
	Keratin Associated Protein 16-8	Krtap16-8	9.45	68484
	Keratin Associated Protein 16-8	Krtap16-9	4.10	170657
