# Supplementary material for: Ablation of Coactivator Med1 Switches the Cell Fate of Dental Epithelia to That Generating Hair
Source: PLoS One. 2014 Jun 20;9(6):e99991. doi: 10.1371/journal.pone.0099991 (PMC4065011; doi:10.1371/journal.pone.0099991)
Supplement: Table S3 — List of genes up-regulated in dental tissues at the Mat stage of Med1 KO (4 wk). Up-regulated genes (p<0.005) are categorized by epidermal differentiation, calcium signaling and cell adhesion involved during development of epidermis. (PDF) [file pone.0099991.s006.pdf]

Table S3

Category	Entrez Gene Name	Symbol	Fold Change	Entrez Gene ID for Mouse
**Transcription factors**	Grainyhead-like 1 (Drosophila)	Grhl1	19.41	195733
	Transcription factor AP-2, gamma	TCFAP2C	3.47	21420
	c-Fos (AP-1)	FOS	1.54	14281
**Cornified envelope**	Corneodesmosin	Cdsn	45.99	386463
	Cornifelin	Cnfn	20.95	72383
	Cornified envelope 1B	Sprrl2	2.76	73722
	Keratinocyte differentiation associated protein	Krtdap	39.95	64661
	Late cornified envelope 1B	Sprrl5	4.24	68720
	Loricrin	Lor	40.69	16939
	Repetin	Rptn	3.00	20129
	Small proline-rich protein 1B	Sprr1b	24.19	20754
	Small proline-rich protein 2D	Sprr2d	3.24	20758
	Small proline-rich-like 7	Sprrl7	2.36	69611
**Epidermal keratins**	Keratin 1	Krt2-1	3.07	6678642
	Keratin 10	Krt10	12.69	16661
	Keratin 15	Krt15	10.96	16665
	Keratin 17	Krt1-17	3.36	16667
	Keratin 23	Krt1-23	64.4	94179
	Periplakin	Ppl	6.55	19041
**Keratinocyte differentiation**	Sciellin	Scel	4.46	64929
	Transglutaminase 1	Tgm1	3.02	21816
	Elongative of very long chain fatty acids like 4	Elovl4	9.87	83603
**Epidermi Lipid Synthesis**	Fatty acid binding protein 5	Fabp5	3.19	16592
**Calcium binding proteins**	S100 Calcium binding protein A7A	S100a15	10.11	381493
	S100 Hornerin	Hrnr	2.97	68723
	Calcium binding epidermal differentiation locus s100a9	S100a9	2.94	20202
	Calcium binding epidermal differentiation locus s100a8	S100a8	3.52	20201
	Calmodulin 4	Calm4	14.62	80796
**Adhesion molecules**	Gap junction beta 2 (connexin 26)	Gjb2	49.81	14619
	Gap junction beta 3	Gjb3	10.82	14620
	Claudin 4	Cldn4	24.82	12740
	Tubulin, beta 2B class IIB	Tubb2b	2.05	73710
