# Supplementary material for: Ablation of Coactivator Med1 Switches the Cell Fate of Dental Epithelia to That Generating Hair
Source: PLoS One. 2014 Jun 20;9(6):e99991. doi: 10.1371/journal.pone.0099991 (PMC4065011; doi:10.1371/journal.pone.0099991)
Supplement: Table S4 — DE-SC signatures are down-regulated in Med1 KO. Genes are categorized by comparison to previous epigenetic analysis of ESC or HF-SC. Genes to regulate tissue specific lineage are shown as ESC because they are bivalent or PGc repressed in ESC. Genes specific for HF are shown as HF-SC and TAC. DE-SC marker candidates that are enriched in CL are shown by fold changes of CL/Mat in CON (left column, red numbers p<0.005). The significant decrease of DE-SC candidates in Med1 KO compared to CON at 10 wk are highlighted by green numbers (right column, fold changes of KO/CON in CL p<0.005). The cellular locations of the genes are also shown beside fold changes. (PDF) [file pone.0099991.s007.pdf]

Table S4

Category	Entrez Gne Name	Symbol	Fold change CL/Mat In CON	Fold change KO/CON in CL	Location	Entrez Gene ID for mouse
**ESC**	sonic hedgehog	**Shh**	15.91	-2.43	Extracellular	20423
	twist homolog 2 (Drosophila)	Twist2	10.62	-0.94	Nucleus	13345
	delta-like 1 homolog (Drosophila)	**Dlk1**	10.16	-0.87	Extracellular	13386
	microfibrillar-associated protein 4	**Mfap4**	9.9	-0.75	Extracellular	76293
	calcium channel, voltage-dependent, T type, a1H	Cacna1h	8.87	-0.09	Membrane	58226
	unc-5 homolog C (C. elegans)	Unc5c	8.61	-0.34	Membrane	22253
	R-spondin 4	RSPO4	8.58	-1.12	Unknown	228770
	matrilin 4 (collagen-like)	**Matn4**	7.86	-0.64	Extracellular	17183
	immunoglobulin superfamily, member 9	Igsf9	7.81	-0.31	Membrane	93842
	keratan sulfate Gal-6 sulfotransferase 1	Chst1	7.68	-0.74	Cytoplasm	76969
	elastin microfibril interfacer 2	Emilin2	7.6	-0.5	Extracellular	246707
	wingless-type MMTV integration site family, 3A	Wnt3a	7.49	-0.36	Extracellular	24117
**HF-SC**	ADAM metallopeptidase with thrombospondin 19	**Adamts19**	10.23	-1.24	Extracellular	240322
	glycoprotein hormone alpha 2	Gpha2	10.11	-0.05	Extracellular	170458
	von Willebrand factor A domain containing 2	**Vwa2**	10.11	-2.2	Extracellular	240675
	protein phosphatase 1, regulatory subunit 3C	Ppp1r3c	9.47	-1.44	Cytoplasm	53412
	sclerostin domain containing 1	Sostdc1	9.39	-1.45	Extracellular	66042
	glypican 3	Gpc3	8.75	-0.88	Membrane	14734
	connective tissue growth factor, Ctgf	**Ctgf**	2.06	-2.75	Extracellular	14219
	proline arginine-rich end leucine-rich repeat	**Prelp**	1.85	-3	Extracellular	116847
	inactive X specific transcripts, Xist	Xist	2.14	-2.96	Nucleus	213742
	cysteine rich protein 61, Cyr61	**Cyr61**	1.61	-3.01	Extracellular	16007
	sox9	Sox9	1.83	-0.89	Nucleus	20682
**TAC**	S100 calcium binding protein A8 (calgranulin B)	S100a8	-	2.35	Cytoplasm	20201
	S100 calcium binding protein A9 (calgranulin B)	S100a9	-	2.73	Cytoplasm	20202
	chitinase 3-like 1 (Chi3l1), mRNA.	Chi3l1		2.17	Extacellular	12654
